# Combining Randomized and Observational Data: The Case of Postnatal Prophylaxis Against HIV

**DOI:** 10.64898/2026.10.15.26365628

**Published:** 2026-10-16

**Authors:** Dwight E. Yin, Paul N. Zivich, Mark J. Giganti, Danni Wu, Jessie K. Edwards, Stephen R. Cole

## Abstract

The efficacy of current antiretroviral interventions for prevention of vertical HIV transmission is both a triumph and a challenge for future efficacy studies of novel agents, including long-acting antiretroviral agents. Given the effectiveness of current antiretroviral interventions, standard randomized controlled trials to assess novel interventions targeted towards further reducing vertical HIV transmission may be infeasible due to required sample sizes. To address this study design challenge, we propose a novel augmented clinical trial design, where a randomized trial with an active comparator is augmented with a concurrent observational cohort to supplement the standard-of-care arm. The proposed design and analysis can overcome sampling biases, and an associated diagnostic can evaluate potential biases from unmeasured covariates. The proposed trial design is illustrated with an example and studied further using a simulation study. In the illustrative example, an augmented trial with a 3:1 allocation ratio in the trial component yielded an unbiased treatment effect estimate, as compared to the trial component alone, and the standard error was reduced substantially. Although the total sample size of the augmented trial was larger than a similarly powered 1:1 standard trial, the sample size of the augmented trial’s trial component was half the size of the standard trial and within the range of historically similar trials, thereby providing both feasibility and potential cost advantages. Although limitations with this design remain, many of these limitations can be mitigated by careful study planning and coordination. Augmented trials provide a potentially applicable solution to studying the efficacy of long-acting antiretrovirals for infant postnatal prophylaxis against HIV.

